# GRK2 Mediates Macrophage Polarization by Regulating EP4-cAMP-pCREB Signaling in Ulcerative Colitis and the Therapeutic Effect of Paroxetine on Mice with DSS-Induced Colitis

**DOI:** 10.3390/ph16050664

**Published:** 2023-04-28

**Authors:** Jiawei Zhang, Xianzheng Zhang, Mingdian Lu, Yan Chang, Qingtong Wang, Jiajie Tu, Huaxun Wu, Chun Wang, Zhongyang Hong, Maoming Xiong, Lihua Song, Wei Wei

**Affiliations:** 1 Institute of Clinical Pharmacology, Anhui Medical University, Key Laboratory of Anti-Inflammatory and Immune Medicine, Ministry of Education, Anhui Collaborative Innovation Centre of Anti-Inflammatory and Immune Medicine, Hefei 230032, China; 2 Department of General Surgery, The First Affiliated Hospital of Anhui Medical University, Hefei 230022, China

**Keywords:** ulcerative colitis, G protein-coupled receptors, macrophage polarization, paroxetine

## Abstract

G protein-coupled receptor kinase 2 (GRK2) is one of the cytosolic enzymes, and GRK2 translocation induces prostaglandin E2 receptor 4 (EP4) over-desensitization and reduces the level of cyclic adenosine monophosphate (cAMP) to regulate macrophage polarization. However, the role of GRK2 in the pathophysiology of ulcerative colitis (UC) remains unclear. In this study, we investigated the role of GRK2 in macrophage polarization in UC, using biopsies from patients, a GRK2 heterozygous mouse model with dextran sulfate sodium (DSS)-induced colitis, and THP-1 cells. The results showed that a high level of prostaglandin E2 (PGE2) stimulated the receptor EP4 and enhanced the transmembrane activity of GRK2 in colonic lamina propria mononuclear cells (LPMCs), resulting in a down-regulation of membrane EP4 expression. Then, the suppression of cAMP–cyclic AMP responsive element-binding (CREB) signal inhibited M2 polarization in UC. Paroxetine is acknowledged as one of the selective serotonin reuptake inhibitors (SSRI), which is also considered as a potent GRK2 inhibitor with a high selectivity for GRK2. We found that paroxetine could alleviate symptoms of DSS-induced colitis in mice by regulating GPCR signaling to affect macrophage polarization. Taken together, the current results show that GRK2 may act as a novel therapeutic target in UC by regulating macrophage polarization, and paroxetine as a GRK2 inhibitor may have therapeutic effect on mice with DSS-induced colitis.

## 1. Introduction

Ulcerative colitis (UC) is known as a chronic complex inflammatory bowel disease that usually involves the sigmoid colon and the rectum with unclear etiology [1]. UC, together with Crohn’s disease (CD), is often identified as an inflammatory bowel disease (IBD). In recent years, the incidence of UC has been showing an increasing trend. The highest incidence of UC is 57.9/100,000 person per year in Northern Europe [2]. Meanwhile, the risk of UC has rapidly increased in newly industrialized nations and is considered as posing a substantial burden to medical systems [3]. To date, a host’s microbiome, genetics, and immune response are all accepted as essential risk factors for the development of UC [4], although the exact pathogenesis of UC is still an unanswered question. The destruction of the intestinal mucosal barrier may be one of the main pathogenetic mechanisms [5]. When the mucosal barrier function has been destroyed, bacterial antigens rapidly invade the colon tissue to activate mucosal immunity and exacerbate the injury of colon.

Monocytes and macrophages play important roles in the innate immune response against invading pathogens. They eliminate pathogens via phagocytosis and by releasing inflammatory mediators, such as cytokines, chemokines, and proteases [6]. Although the sources and phenotypes of macrophages are not the same everywhere, they all maintain a dynamic balance. The destruction of this balance may cause various diseases. Under the induction of a variety of factors, polarized macrophages can form different functional phenotypes: classical M1-type macrophages with proinflammatory functions and M2-type macrophages with anti-inflammatory functions [7]. M1 macrophages are characterized by Th1-type immune response and secretion of pro-inflammatory cytokines, whereas M2 macrophages are involved in Th2-type immune response. As a consequence, inappropriate activation of macrophage polarization can induce sustained inflammation, resulting in autoimmune and inflammatory diseases. In our previous study, an analysis of colon tissue samples from UC patients showed the infiltration of macrophages. The M1/M2 ratio presented in the intestinal mucosa markedly increased in UC. Thus, regulation of the balance of M1/M2 may be important for the treatment of UC [8].

G protein-coupled receptor kinases (GRKs) are one large group of kinases that cause rapid desensitization after binding to GPCRs. They belong to the serine/threonine protein kinase family and are considered to be important negative regulatory proteins in the GPCR signaling pathway [9]. GRKs are also widely distributed in digestive tract tissues [10]. Prostaglandin E2 (PGE2) is an inflammatory mediator that plays an essential role in the occurrence and development of UC [11]. A previous study found that PGE2 could increase the level of cAMP in the cytoplasm, further activated CREB, and then promoted the polarization of M2 macrophages [12]. GRK2 is considered an important negative regulatory protein in the GPCR signaling pathway. Due to the influence of the polarization of macrophages, regulation of GRKs may be a new therapeutic strategy for UC.

Paroxetine, a representative of serotonin reuptake inhibitors, is often used for treating mental disorders, such as generalized anxiety disorder and postpartum depression, and has fewer side effects than first-generation selective 5-HT reuptake inhibitors [13]. Previous studies have demonstrated that selective 5-HT reuptake inhibitors have therapeutic effects on UC patients with severe emotional problems [14]. Gut–brain axis dysregulation has been recognized as having a crucial role in our understanding of chronic gastrointestinal diseases. Recently, IBD is considered a disorder involving a multi-crosstalk pathway with the gut–brain axis [15]. Some studies showed that paroxetine could alleviate psychological comorbidities to improve IBD patients’ quality of life [16]. Encouragingly, Ning L. et al. reported that paroxetine has therapeutic effect on intestinal inflammation by controlling microorganisms in the digestive tract [17].

Paroxetine has also been identified as a direct inhibitor of GRK2, and it has been extensively reported to have benefits in osteoarthritis, cardiac hypertrophy, myocardial infarction, and cutaneous anaphylaxis [18,19,20,21]. In recent studies, GRK2 was reported to be a critical regulator of TNFα-induced MAPK and NF-κB pathways in intestinal epithelial cells [22], and GRK2-deficient mice were protected from DSS-induced acute colitis [23]. Thus, we hypothesized that paroxetine may have therapeutic effects by regulating GRK2 signals in IBD.

The effect of GRK2 on the polarization of macrophages by regulating downstream signaling pathways is still unknown in UC. In this study, we investigated how GRK2 influences the polarization of macrophages via the PGE2-EP4-cAMP-pCREB signaling pathways in UC patients. Using DSS-induced colitis mice and the THP-1 cell line, we demonstrated that GRK2 is involved in the polarization of macrophages and provided evidence for the new mechanism of paroxetine in the treatment of UC.

## 2. Results

### 2.1. Macrophage Polarization in UC

**Figure 1:**
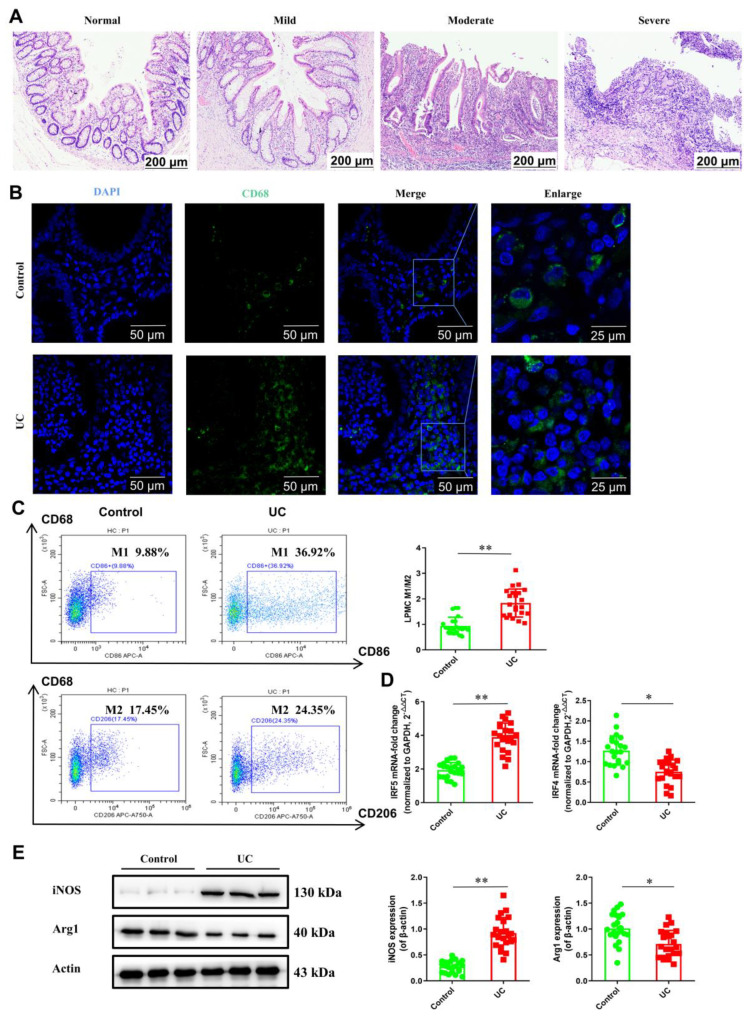
Macrophage polarization in UC. (**A**) Representative H&E staining images of colon tissue (scale bar, 200 μm). (**B**) Confocal imaging of CD68 in colon tissue using fluorescence microscope (scale bar, 50 μm). (**C**) The percentages of M1 and M2 in LPMCs were determined using flow cytometry (M1: CD68^+^/CD86^+^; M2: CD68^+^/CD206^+^). (**D**) mRNA expressions of IRF5 and IRF4 were detected using RT−PCR. (**E**) The protein levels of iNOS and Arg1 were determined using Western blot. Data are represented as mean ± SEM (*n* = 22). Significant differences are indicated as * *p* < 0.05 and ** *p* < 0.01 versus the control group using *t*-test.

From the representative H&E staining images of the UC colon tissue, we found evidence of damage from the destruction of epithelial structures and surface contour changes, including villiform contours, loss of crypts, and crypt abscess (Figure 1A). To investigate the density of macrophages in the UC colon tissue, macrophages were labeled with CD68, and the expression difference between the control group and the UC group was observed using a fluorescence microscope (Figure 1B). The results showed that the expression of CD68 in the colonic lamina propria of the UC group was significantly stronger than the control group. We then further investigated the proportion of M1 macrophages and M2 macrophages, which have been found to play important roles in the development of colitis. The results showed that in the UC group, the infiltration of M1 macrophages was significantly higher than M2 macrophages in the LPMCs. Compared to the control group, the M1/M2 ratio increased significantly in the UC group (Figure 1C). Meanwhile, we analyzed the gene expression of M1/M2 specific factors in the LPMCs of the UC patients. The results showed that mRNA expression of M1 macrophage-associated factor IRF5 was significantly increased, while the level of M2 macrophage-associated marker IRF4 was markedly decreased, in the UC group (Figure 1D). The Western blot (WB) revealed that iNOS expression in the UC group was greatly increased and Arg-1 expression was reduced (Figure 1E). Taken together, these results imply that the balance between M1 and M2 macrophages is interrupted in UC patients.

### 2.2. GRK2 Mediates the Activation of PGE2-EP4-cAMP-pCREB Pathway in Colonic LPMCs of UC Patients

**Figure 2:**
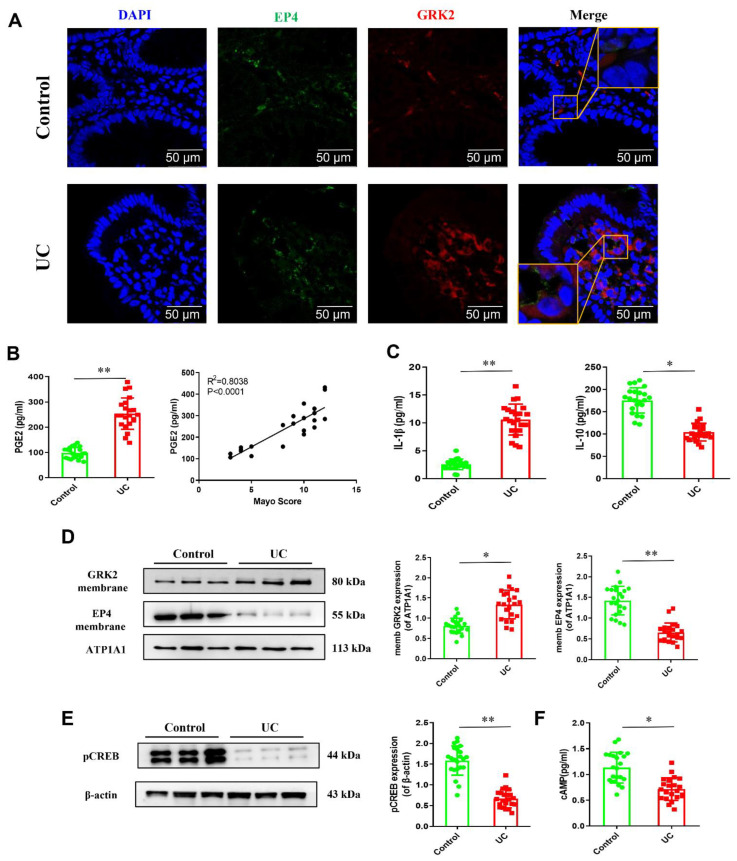
GRK2 mediates changes in the PGE2−EP4−cAMP−pCREB pathway in colonic LPMCs of UC patients. (**A**) Confocal imaging and co−localization of GRK2 and EP4 in colon tissue (scale bar, 50 μm). (**B**) The level of PGE2 in LPMCs and the Spearman correlation between the Mayo score and PGE2 level. (**C**) The levels of IL−1β and IL−10 in LPMCs were determined using ELISA. (**D**) The membrane protein levels of GRK2 and EP4 in LPMCs were determined using Western blot. (**E**) Representative band of pCREB. (**F**) The level of cAMP in LPMCs was determined using ELISA. Data are represented as mean ± SEM (*n* = 22). Significant differences are indicated as * *p* < 0.05 and ** *p* < 0.01 versus the control group using *t*-test.

Firstly, we investigated the co-localization of GRK2 and EP4 in colon tissue. We used 488 nm green fluorescent labeling for EP4 and 594 nm red fluorescent labeling GRK2. The immunofluorescence showed that GRK2 and EP4 were mainly distributed on the membrane and cytoplasm, according to the Merge diagram, in the UC group, and the co-binding of GRK2 and EP4 was significantly increased on the membrane compared with the control group (Figure 2A). As shown in Figure 2D, the membrane expression of GRK2 increases remarkably in the colonic LPMCs of the UC group. Conversely, the membrane expression of EP4 decreases, suggesting that increased GRK2 transmembrane leads to the down-regulation of EP4 receptors. The ELISA results indicated that the level of PGE2 increased in the colonic LPMCs of the UC group, and the trend had a significant positive correlation with the Mayo score (*p* < 0.0001, Figure 2B). The levels of pro-inflammatory cytokines, such as IL-1β, were also elevated. In contrast, the levels of anti-inflammatory cytokines, such as IL-10, dropped in the UC group (Figure 2C). To further determine whether cAMP-pCREB signaling rebalanced the macrophage polarization, we detected cAMP in LPMCs using ELISA. The result showed that cAMP decreased significantly in the UC group (Figure 2F). The WB showed that the expression of pCREB also decreased in the LPMCs (Figure 2E). Therefore, we speculated that the high level of PGE2 stimulated the receptor EP4 and enhanced the transmembrane activity of GRK2 in LPMCs, resulting in the down-regulation of EP4 membrane expression.

### 2.3. GRK2 Heterozygous Mice Are Protected from DSS-Induced Colitis

**Figure 3:**
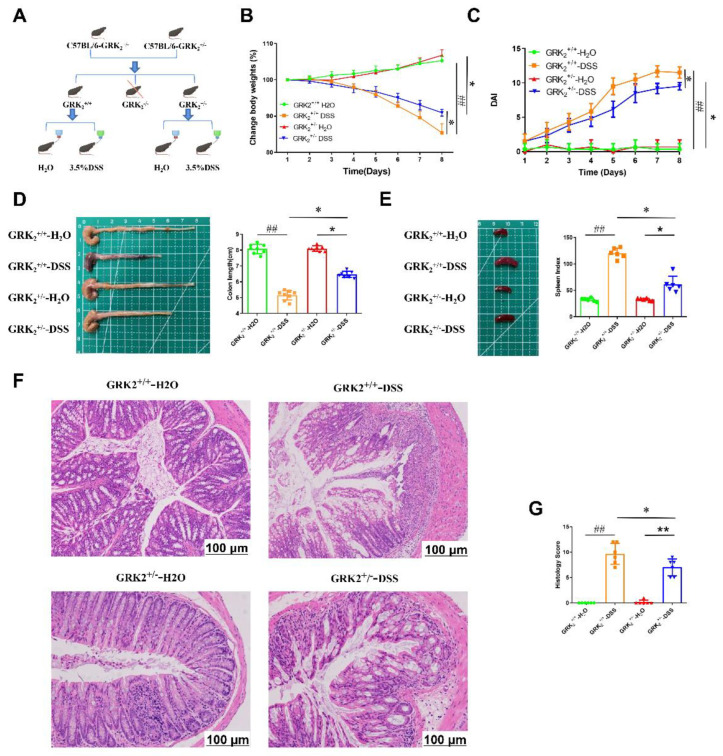
GRK2 heterozygous mice are protected from DSS−induced colitis. (**A**) Experimental design for DSS−induced colitis in WT littermates and GRK2 heterozygous mice. (**B**) Body weight changes and (**C**) disease activity index (DAI). (**D**) Macroscopic photographs of the colons and length of the colons. (**E**) Macroscopic photographs of the spleen and spleen index. (**F**) Representative H&E staining images of colon tissue (scale bar, 100 μm). (**G**) Histological scores of colon tissue. Data are represented as mean ± SEM (*n* = 6). Statistical analysis was performed using two−way ANOVA with Dunnett’s Multiple Comparison test. Significant differences are indicated as * *p* < 0.05 versus the GRK2^+/+^-DSS group; * *p* < 0.05 and ** *p* < 0.01 versus the GRK2^+/−^-H_2_O group; and ^##^ *p* < 0.01 for the GRK2^+/+^-DSS group versus the GRK2^+/+^-H_2_O group.

In order to investigate the role of GRK2 in the progression of colitis, we selected GRK2^+/−^ heterozygous and WT littermates. Then, we established a mouse model via feeding with 3.5% DSS solution for seven consecutive days (Figure 3A). Following the 3.5% DSS treatment, the GRK2^+/+^ mice lost significant body weight beginning at day 3 and displayed signs of severe disease, such as loose and bloody stool, ruffled hair, and hunched posture. Compared to the GRK^+/−^-DSS group, the mice in the GRK2^+/+^-DSS group lost their bodyweight significantly (Figure 3B) and had higher DAI scores (Figure 3C). DAI is a key indicator to evaluate the severity of colitis, including weight loss, rectal bleeding, and stool consistency. Moreover, the GRK2^+/−^-DSS mice had longer colons and lower spleen index than the GRK2^+/+^-DSS mice (Figure 3D,E). The H&E staining of the colons showed that compared to the GRK2^+/−^-DSS mice, the GRK^+/+^-DSS mice had a more serious situation of loss of epithelial crypts, structural disorders, extensive infiltration of inflammatory cells in the submucosa (Figure 3F), and higher pathological score (Figure 3G). These results showed that the GRK2^+/−^ mice were significantly protected from DSS-induced colitis.

### 2.4. GRK2 Regulates Macrophage Polarization through PGE2-EP4-cAMP-pCREB Pathway in DSS-Induced Colitis Model

**Figure 4:**
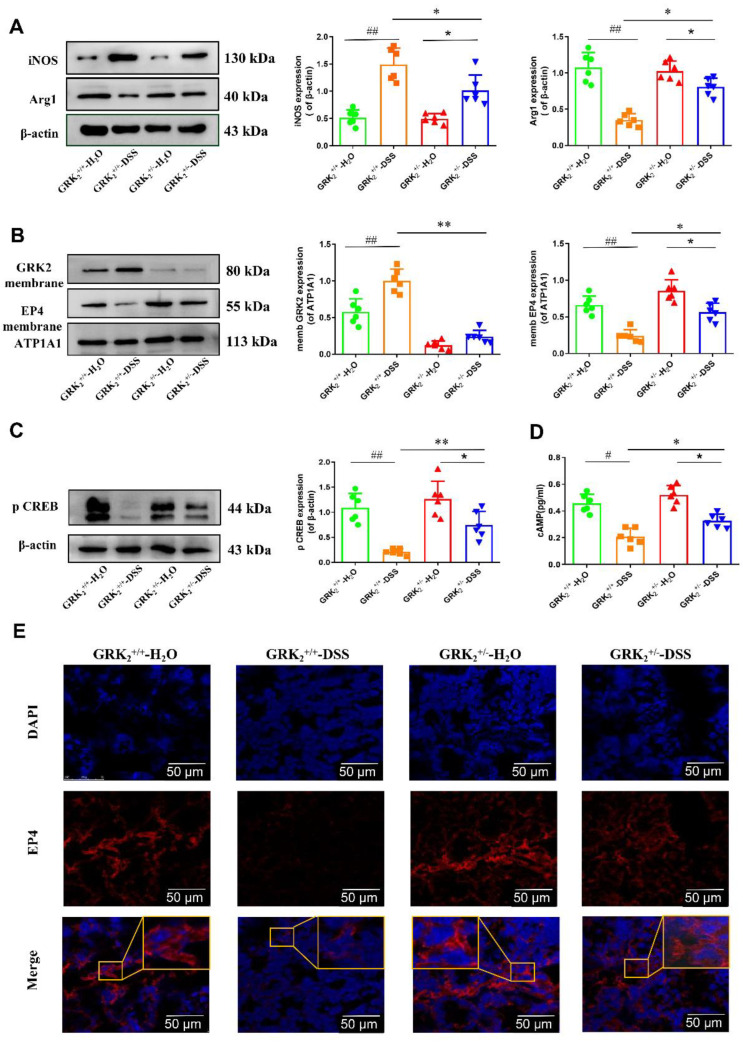
GRK2 regulates macrophage polarization via the PGE2−EP4−cAMP−pCREB pathway in DSS−induced colitis model. (**A**) The protein levels of iNOS and Arg1 in LPMCs were determined using Western blot. (**B**) The membrane protein levels of GRK2 and EP4 were determined in LPMCs using Western blot. (**C**) Representative band of pCREB. (**D**) The level of cAMP in LPMCs was determined using ELISA. (**E**) Confocal imaging of EP4 (red) in colon tissue using fluorescence microscope (scale bar, 50 μm). Data are represented as mean ± SEM (*n* = 6). Statistical analysis was performed using two-way ANOVA with Dunnett’s Multiple Comparison test. Significant differences are indicated as * *p* < 0.05 and * *p* < 0.01 versus the GRK2^+/+^−DSS group; * *p* < 0.05 and ** *p* < 0.01 versus the GRK2^+/−^-H2O group; and ^#^ *p* < 0.05 and ^##^ *p* < 0.01 for the GRK2^+/+^-DSS group versus the GRK2^+/+^-H2O group.

To determine GRK2-dependent regulation of macrophage polarization, we collected fresh mouse colon tissue to extract LPMCs when the mice were euthanized on day 8. Then, we examined the level of protein expression using WB and found that compared to the GRK2^+/+^-DSS group, iNOS expression greatly decreased and Arg-1 expression increased in the GRK2^+/−^-DSS group (Figure 4A). We investigated the impact of GRK2 knockout on the EP4-cAMP-pCREB pathway in the DSS-induced colitis model, and the level of cAMP was found to decrease significantly in the GRK2^+/+^-DSS group (Figure 4D). Furthermore, the WB results showed that compared to the GRK2^+/+^-DSS group, the EP4 membrane expression and pCREB expression increased significantly in the GRK2^+/−^-DSS group (Figure 4B,C). We further investigated the EP4 expression difference between the GRK2^+/+^-DSS group and the GRK2^+/−^-DSS group using a fluorescence microscope (Figure 4E). The results showed that the expression of EP4 in the LPMCs of the GRK2^+/−^-DSS group was significantly stronger than in the GRK2^+/+^-DSS group.

**Figure 5:**
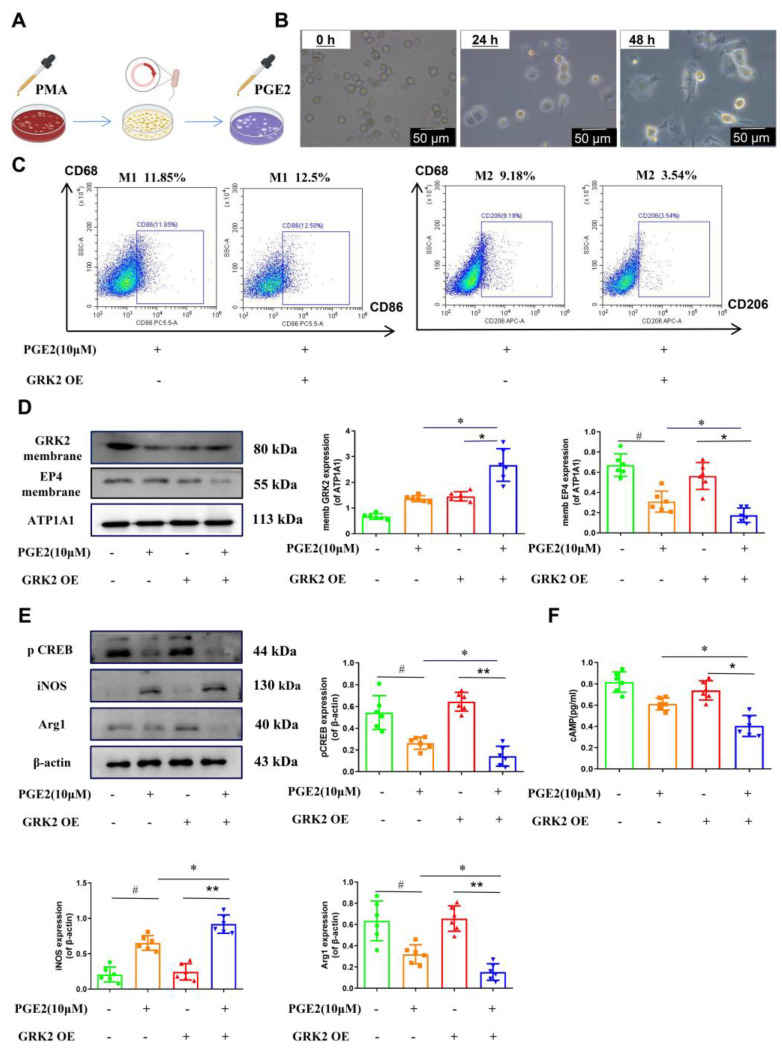
Macrophage polarization in THP−1 differentiated macrophages (THP−Ms) after transfected with pIRES−EGFP−GRK2−WT. (**A**) Experimental design for THP−1 differentiated macrophages (THP−Ms) which were then transfected with plasmid. (**B**) Microscopic image of THP−1 cells treated with PMA (100 ng/mL) for 48 h to differentiate into macrophages (THP−Ms) (scale bar, 50 μm). (**C**) The percentages of M1 and M2 in THP−Ms were determined using flow cytometry (M1: CD68^+^/CD86^+^; M2: CD68^+^/CD206^+^). (**D**) The membrane protein levels of GRK2 and EP4 were determined using Western blot. (**E**) Representative bands and protein levels of pCREB, iNOS, and Arg1 were detected using Western blot. (**F**) The level of cAMP in THP−1 differentiated macrophages (THP−Ms) was determined using ELISA. Data are represented as mean ± SEM (*n* = 3). Statistical analysis was performed using two−way ANOVA with Dunnett’s Multiple Comparison test. Significant differences are indicated as * *p* < 0.05 and ** *p* < 0.01 versus GRK OE^−^ group with the stimulation with PGE2 (10 μM); * *p* < 0.05 and ** *p* < 0.01 versus GRK OE^+^ group without the stimulation with PGE2 (10 μM); and ^#^ *p* < 0.05 for the GRK OE^−^ group without the stimulation with PGE2 (10 μM) versus the GRK OE^−^ group with the stimulation with PGE2 (10 μM).

As can be seen from the above results, GRK2 may be a key factor affecting macrophage polarization. To further explore the impact of altered GRK2 protein activity on GRK2-mediated EP4 down-regulation and cAMP-pCREB activation, we chose THP-1 cells, which were stimulated with PMA (100 ng/mL) for 48 h to induce M0 macrophages (Figure 5B) and then transiently transfected with pIRES-EGFP-ctr and pIRES-EGFP-GRK2-WT plasmids (Figure 5A). The fluorescence microscopic results showed that GRK2 plasmid was successfully expressed in THP-1 cells. With the stimulation using PGE2 (10 μM) for 30 min, the percentage of M2 (CD206^+^CD68^+^) decreased significantly in the GRK2 overexpressed group compared to the non-overexpressed group (Figure 5C). Meanwhile, in the GRK2 overexpressed group, the iNOS level increased and the Arg-1 and pCREB levels decreased significantly compared to the non-overexpressed group (Figure 5E). After the stimulation with PGE2 (10 μM), EP4 membrane expression decreased significantly in the GRK2 overexpressed group compared to the non-overexpressed group (Figure 5D). The ELISA results showed that the cAMP level also had the same trend (Figure 5F). These results suggest that GRK2 translocation to the membrane is related to EP4 down-regulation, and GRK2 maybe a key factor affecting macrophage polarization via the PGE2-EP4-cAMP-pCREB pathway.

### 2.5. Paroxetine Has Therapeutic Effect on Mice with DSS-Induced Colitis by Inhibiting GRK2 Translocation to Regulate Macrophage Polarization

The chemical structure of paroxetine is presented in the Supplementary Figure S1A. We further explored whether paroxetine attenuated symptoms of DSS-induced colitis in mice and the differences in therapeutic effect between paroxetine and SASP (the drug commonly used for IBD). We established the mouse model by feeding the mice with 3.5% DSS for seven consecutive days. Compared to the control group, the mice in the DSS group lost bodyweights significantly, whereas paroxetine and SASP as the positive controls rescued the loss of body weights (Supplementary Figure S1B). The disease activity index (DAI), which is one important parameter reflecting the severity of colitis, significantly decreased after the administration of paroxetine (Supplementary Figure S1C). As representative markers of colitis, colonic length (Supplementary Figure S1D) and splenomegaly (Supplementary Figure S1E) were found in all DSS groups, which were rescued to varying degrees by paroxetine. In addition, the histopathological evaluation revealed that DSS-elicited colonic inflammation, including crypt abscess, infiltration of inflammatory cells, and disruption of the mucosal barrier, all directly led to a higher histological score (Supplementary Figure S1F,G). By contrast, either paroxetine or SASP administration improved the pathological destruction and decreased the higher histological scores of the DSS-induced colitis mice. Taken together, our results revealed that paroxetine could alleviate the symptoms of DSS-induced colitis in mice.

**Figure 6:**
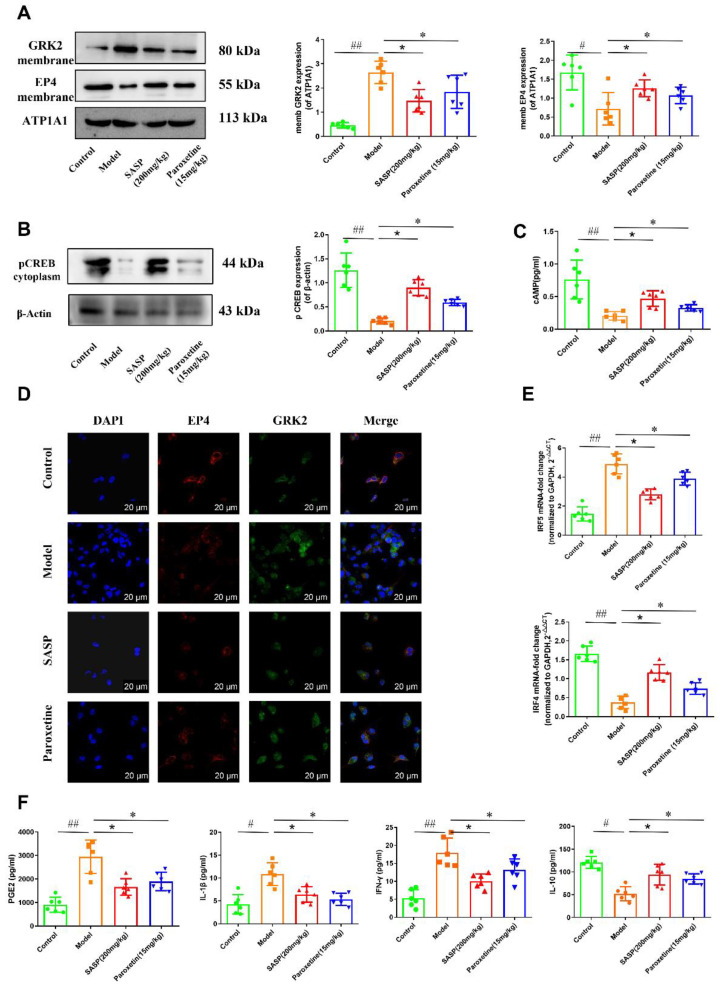
Paroxetine down−regulates EP4/cAMP/pCREB−dependent GRK2 translocation in mice with DSS−induced colitis. (**A**) The membrane protein levels of GRK2 and EP4 in the mice’s LPMCs were analyzed using Western blot. (**B**) The protein level and representative band of pCREB. (**C**) The level of cAMP in LPMCs was determined using ELISA. (**D**) Confocal imaging and co−localization of GRK2 (green) and EP4 (red) in LPMCs based on fluorescence microscope (scale bar, 20 μm). (**E**) mRNA expressions of IRF5 and IRF4 were detected using RT−PCR. (**F**) The levels of PGE2, IL−1β, IFN−γ, and IL−10 in LPMCs were determined using ELISA. The data are presented as the means ± SEM (*n* = 6). Statistical analysis was performed using one−way ANOVA with Dunnett’s Multiple Comparison test. Significant differences are indicated as ^#^ *p* < 0.05 and ^##^ *p* < 0.01 vs. control group; * *p* < 0.05 vs. paroxetine−treated group; and * *p* < 0.05 vs. SASP−treated group.

To confirm whether paroxetine affected GRK2 translocation in mice with DSS-induced colitis, we first detected the expression of GRK2/EP4/cAMP/pCREB in the mice’s LPMCs. Compared to the control group, GRK2 membrane expression increased significantly, and EP4 membrane expression and pCREB level in cytoplasm decreased in the DSS groups. Then, the expression of pCREB in cytoplasm and EP4 membrane expression also significantly increased after being treated with paroxetine. (Figure 6A,B). Compared to the DSS groups, the ELISA results showed that the cAMP level increased significantly in the paroxetine-treated group (Figure 6C). Additionally, we noted that the expression of membrane GRK2 in LPMCs was down-regulated, and the expression of membrane EP4 was up-regulated and inhibited the membrane association of GRK2 and EP4 in the paroxetine-treated group (Figure 6D).

In IBD, the ratio of M1/M2 is often associated with the development of inflammatory disorders. To investigate the changes in the ratio of M1/M2, PMs were isolated from the mice with DSS-induced colitis and evaluated using qRT-PCR. The results indicated that mRNA expression of M1 macrophage-associated factor IRF5 was significantly increased, while the level of IRF4 (M2 macrophage-associated markers) was markedly decreased in the model group (Figure 6E). Both paroxetine and SASP administration increased the percentage of M2 markedly in the DSS-induced colitis mice. In order to detect whether paroxetine could regulate PGE2, INF-γ, IL-1β, and IL-10 levels in mice with DSS-induced colitis, ELISA was used to examine the levels of PGE2, INF-γ, IL-1β, and IL-10 in LPMCs. In all DSS groups, PGE2, INF-γ and IL-1β showed high levels of expression and IL-10 showed a lower level of expression (Figure 6F). Compared to the model group, PGE2, INF-γ, and IL-1β expression levels reduced following the treatment with paroxetine. These results indicate that paroxetine can increase M2 percentages and regulate corresponding inflammatory cytokine production in mice with DSS-induced colitis.

## 3. Discussion

Recently, numerous research studies on UC have identified the importance of macrophages in the pathogenesis of UC [24,25]. Macrophages are also widely distributed in the gut and often play an important role in different kinds of physiological processes, including acute and chronic inflammation, and pathogen defense [26]. Macrophage polarization can be broadly classified into two main groups: classically activated macrophages and alternatively activated macrophages, which induce proinflammatory responses and anti-inflammatory regulation. These macrophages are called M1 and M2 and induce iNOS or arginase, respectively. The M1/M2 imbalance plays a pathogenic role in autoimmune diseases [27].

In our study, the UC patients’ colon mucosal lesions were characterized based on the infiltration of inflammatory cells, which mainly were identified macrophages. It has been reported that the ratio of M1/M2 increases in UC [28]. In the present study, we found the same trend using flow cytometry. These data show that the imbalance of M1/M2 may be related to the pathogenesis of UC. PGE2 has been proven to be an important immune mediator, and it always has a function in autocrine and paracrine signaling. One study indicated that a high level PGE2 could promote M2 polarization through the cAMP-CREB pathway [29]. The exact function of PGE2 in inflammatory diseases remains controversial. A previous research study of our group showed that constant stimulation of PGE2 on fibroblast-like synovial cell (FLS) of CIA rats resulted in a decreased level of cAMP, which was primarily caused by GRK2-induced EP4 over-desensitization [30]. The decreased level of cAMP inhibited the production of IRF4 transcription factor, eventually affecting the polarization to M2-type macrophages. From our previous study, the PGE2 treatment of HUVECs increased the phosphorylation of GRK2-Ser685, which then influenced the membrane (memb) and cytoplasm (cyt) expressions of EP4 and GRK2. These results indicated that the stimulation of PGE2 could cause GRK2-induced EP4 over-desensitization [31].

**Figure 7:**
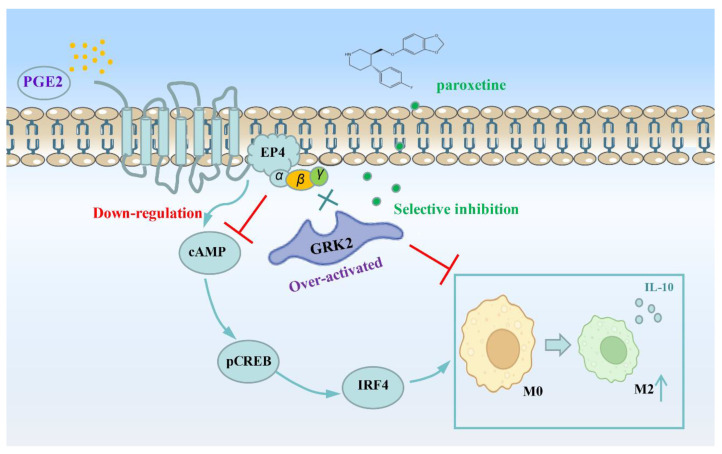
The proposed model shows some of the potential translocation activity-dependent mechanisms by which GRK2 may modulate EP4-cAMP-pCREB signaling to regulate macrophage polarization in colitis and the alleviation after treatment with paroxetine. Under the condition of inflammation, PGE2 binds to ligand EP4 receptor, mediating cAMP/pCREB signaling activation to enhance M2 polarization. Similarly, membrane targeting of over-activated GRK2 is associated with EP4 receptor, and this maintains persistent EP4 receptor down-regulation to influence M2 polarization. Paroxetine can inhibit GRK2 membrane recruitment to recover the balance in GPCR signaling and, thus, alleviate inflammatory response in colitis.

GRK2 is known as one of the serine/threonine protein kinases. After GRK2 specifically recognizes GPCR, it then mediates phosphorylation and regulates GPCR desensitization. GPCR desensitization could mediate downstream signal transduction to regulate physiological function. Our group has long been committed to the role of GRK2 in RA and arthritis animal models. We investigated the mechanism of GRK2 in macrophage polarization in RA [32]. We found that the phenomenon of membrane localization of GRK2 increased in macrophages in UC, as well as increased the combination between EP4 and GRK2. Therefore, GRK2-induced down-regulation of EP4 membrane expression may be the pathogenesis of ulcerative colitis (Figure 7).

In this work, we found that GRK2-mediated PGE2-EP4-cAMP-pCREB signaling induced an imbalance in the ratio of M1/M2 in UC for the first time. First, we found that the secretion of PGE2 was increased in LPMCs of the UC group, and this trend had a significant positive correlation with the Mayo score. Second, to detect the role of GRK2 on the down-regulation of EP4 membrane expression, we used laser confocal microscopy and found that the interaction between GRK2 and EP4 increased significantly in the UC group. The results regarding the membrane expressions of GRK2 and EP4 are consistent with those of the laser confocal microscopic experiment.

Next, to illuminate the role of GRK2 on macrophage polarization, we stimulated the THP-1 cell line with 100 ng/mL of PMA for 48 h consecutively, which then transformed into M0 macrophages. After pIRES-EGFP-GRK2 plasmids were successfully constructed and transfected, PGE2 (10 μM) was used to stimulate M0 macrophages transfected with GRK2-overexpressed plasmids, and the ratio of M1 (CD68^+^CD86^+^)/M2 (CD68^+^CD206^+^) was detected using flow cytometry. The results showed that under the same stimulation conditions, the ratio of M1/M2 increased significantly in the pIRES-EGFP-GRK2 group compared to the control group. Additionally, the expression of iNOS increased and the level of Arg1 decreased, suggesting that GRK2 is involved in the regulation of macrophage polarization.

Then, we generated GRK2 heterozygous mice (GRK^+/−^) to explore the role of GRK2 in experimental colitis. Compared to the GRK^+/+^ mice, membrane localization of EP4 significantly increased in the GRK^+/−^ mice. At the same time, the ratio of M1/M2 in the GRK^+/−^ mice decreased, and the degree of pathological damage of colon tissue was lower. All these results indicated that the reduction in GRK2 contributed to restoring the membrane expression of EP4 and then rebalancing the ratio of M1/M2 via cAMP-pCREB signaling.

Symptoms of generalized anxiety disorder and various degrees of depression are very common in IBD. Different antidepressants are taken by approximately 30% of people with IBD [33]. Previous studies have found that selective serotonin reuptake inhibitors (SSRI) play a protective role in severe UC patients by managing physical symptoms, but the data are low-certainty evidence [34,35]. Recently, Ning L. et al. reported that paroxetine has therapeutic effect on a DSS-induced colitis mouse model by controlling microorganisms in the digestive tract, and the results indicate an interesting phenomenon that fluoxetine (another frequently used SSRI) shows no statistically significant associations with DAI scores, bowel length, body weight, and pathology scores [17]. Paroxetine, an FDA-approved SSRI, was identified as a potent GRK2 inhibitor with a higher selectivity for GRK2 over other GRKs both in vivo and in vitro [36]. Paroxetine binds to the active site of GRK2 to inhibit GPCR phosphorylation. Paroxetine can inhibit GRK2 membrane recruitment to recover macrophage polarization by regulating GPCR signaling (Figure 7).

## 4. Materials and Methods

### 4.1. Patients and Clinical Score

The present study was approved by the Research Ethics Committee of the First Affiliated Hospital of Anhui Medical University (No. 5101449, Hefei, China), and written informed consents were provided by all patients. This study was performed following the Declaration of Helsinki. After providing informed consent, a total of 22 patients with UC (UC group) and 22 volunteers who had received colonoscopy (Control group) at the First Affiliated Hospital of Anhui Medical University (Hefei, China) between January 2019 and December 2021 were recruited into this research. The participants had complete clinical characteristic (Supplementary Table S1) and laboratory examination data (Supplementary Table S2). The patients were diagnosed based on endoscopic and histological criteria. The inclusion criteria for this study were UC diagnosed patients, aged 18–75 years old, and no history of gastrointestinal surgery. The exclusion criteria for this study were patients with non-classifiable IBD, indeterminate colitis, or infectious colitis, and patients who had undergone gastrointestinal surgery. Colon specimens and peripheral blood were obtained from the UC patients and volunteers. The disease activity index of the patients was obtained based on the Mayo score for UC.

### 4.2. Materials

Paroxetine was purchased from Abcom (Lot#: ab120069, Cambridge, UK). Salicylazosulfapyridine (SASP, Lot#: 230014) was purchased from the Shanghai XinYi Pharmaceutical Co., Ltd. (Shanghai, China). Dextran sulfate sodium (DSS, MW: 36000–50000, Lot#: 02160110-CF) was obtained from MP Biomedicals (Irvine, CA, USA). Antibodies for GRK2 (80 Kd, Lot#: Sc-13143) and EP4 (55 Kd, Lot#: Sc-55596) were purchased from Santa Cruz (Rosemont, IL, USA). Antibodies for iNOS (131 kd, Lot#: GTX130246) were purchased from GeneTex (Rosemont, IL, USA). Antibodies for Arg-1 (40 Kd, Lot#: 93668) and pCREB (43 Kd, Lot#: 9198) were purchased from Cell Signaling Technology Co., Ltd. (Erie, PA, USA). cAMP Activity Assay Kit was obtained from BioVision Inc (San Francisco, CA, USA). Antibodies for β-actin (Lot#: E021020), anti-rabbit IgG (Lot#: E030120), and anti-mouse IgG (Lot#: E030110) were obtained from EarthOx Life Sciences (Burlingame, CA, USA). Alexa Fluor 488- and Alexa Fluor 594-tagged secondary antibodies were purchased from Proteintech (Chicago, IL, USA). Serum levels of PGE2, IL-1β, IL-10, and IFN-γ were determined using an ELISA kit following the manufacturer’s protocol (Enzyme-linked Biotechnology Co., Ltd., Shanghai, China). All other reagents and chemicals were of commercially available analytical grade.

### 4.3. Histological Analysis

We used 4% paraformaldehyde to fix the colon specimens overnight and then used paraffin to embed them. The tissues were cut into 4 μm thick sections for routine hematoxylin and eosin (H&E) staining. Two pathologists, who were blinded to this study, evaluated all sections, and then we captured the images using an optical microscope (OLYMPUS, Tokyo, Japan).

### 4.4. Immunofluorescence Assay of Colonic Mucosal Tissues

We first used fresh colonic mucosal tissues to prepare frozen sections (Leica, CM1950, Germany). Then, the sections were permeabilized by Triton-X-100 (Beyotime) for 7 min, blocked with 10% bovine serum (CWBIO) for 30 min, and finally incubated with monoclonal anti-GRK2 (80 Kd, Lot#: Sc-13143) and anti-EP4 antibodies (55 Kd, Lot#: Sc-55596) overnight at 4 °C in a wet chamber. IgG antibody was used as a negative control to check that non-specific interaction of the primary antibody did not cause the observed staining. After being washed with PBS, the samples were incubated with Alexa Fluor 488- and Alexa Fluor 594-tagged second antibodies at 37 °C for 1 h and subsequently incubated with DAPI (10 μg/mL) for 7 min. The images were captured using a TCS SP8 confocal microscope (Leica Microsystems, Wetzlar, Germany).

### 4.5. Preparation of Lamina Propria Mononuclear Cells (LPMCs) from Colonic Mucosa

LPMCs were isolated from surgically resected colon tissues using an enzymatic technique [37]. Firstly, we used normal saline to remove blood and feces from the colon tissue surface. Then, we dissected the mucosa with HBSS (Gibco, Carlsbad, CA, USA) supplemented with 1 mM of dithiothreitol (Sigma, St. Louis, MO, USA) to remove mucus. Secondly, we used sterile ophthalmological scissors to cut the mucosa into crumbs and then incubated them for 2 h in a PBS culture medium (Thermo, South Logan, UT, USA) with 0.5 mg/mL of type IV collagenase and 0.5 mg/mL of DNase I (Roche, Indianapolis, IN, USA). Finally, we separated the cellular fraction once using a 10% red blood cell lysate solution, and the target cells were centrifuged using the Ficoll–Hypaque density gradient (GE, Uppsala, Sweden). The target LPMC population was collected using flow cytometry.

### 4.6. Animals

GRK2 heterozygous mice on the C57BL/6J background were obtained from GemPharmatech (Nanjing, China). The GRK2^+/−^ heterozygous and WT littermates (20 ± 2 g) were used for the indicated experiments and were bred at the experimental animal center of Anhui Medical University (Hefei, China). The animals were housed in a specific pathogen-free environment at a temperature of 23 ± 2 °C and humidity of 55 ± 10% with a 12 h light/dark cycle and were given standard laboratory diet and water. The experimental protocols were approved by the Animal Experimental Ethics Committee of Anhui Medical University (No. 20220132) and strictly followed the ethical regulation of the Committee for Animal Care and Use at Anhui Medical University and the Guide for the Care and Use of Laboratory Animals (NIH, Bethesda, MA, United States). All efforts were made to minimize the animals’ suffering and to reduce the number of animals used. The animals were sacrificed using cervical vertebra luxation, and then sealed and stored in an animal carcass dedicated freezer.

### 4.7. Experimental Groups

The sample size was determined using the G*Power 3.1 software (3.1.5.7; HeinrichHeine-Universität Düsseldorf, Düsseldorf, Germany). Based on the differences in the parameters between the control and DSS-induced acute colitis mice, we calculated the sample size required for each group to reach a 5% significance and 0.80 power. The analysis showed that at least five mice in each group were required. Thus, we increased the number of mice to 6 to account for any accidental side effects due to the treatments.

In order to detect whether the GRK2-deficient mice were protected from DSS-induced acute colitis, after acclimatizing for at least one week, the GRK2^+/−^ heterozygous mice and WT mice (20 ± 2 g, male) were randomly divided into 4 groups (*n* = 6): GRK2^+/+^-H2O group, GRK2^+/+^-DSS group, GRK2^+/−^-H2O group, and GRK2^+/−^-DSS group.

To understand whether paroxetine could alleviate DSS-induced acute colitis in mice, 6-week-old male wild-type C57BL/6 mice were divided into following groups (*n* = 6): control group (PBS) and model group (3.5% DSS). Sulfasalazine (200 mg/kg per day + 3.5% DSS) and paroxetine (15 mg/kg per day + 3.5% DSS) were administered daily by gavage. After harvest, the colons were collected and their length was measured, and the colons were then fixed with formalin and embedded in paraffin for further analyses.

### 4.8. Establishment of DSS-Induced Colitis Mouse Model and Evaluation of Disease Activity Index (DAI)

After acclimatizing for at least one week, experimental colitis was induced in the mice by administrating 3.5% dextran sodium sulfate for 7 consecutive days as previously described [38], while the control mice were given the same volume of distilled water. During the 3.5% DSS treatment, the mice were examined daily and scored for disease activity index using specific criteria (Supplementary Table S3). At the time of harvest, splenic weight and colon length were additionally measured.

### 4.9. Histopathological Assessment

At harvest, we first removed the fecal content, and then the colon tissues were dehydrated and embedded in paraffin after being fixed in 4% paraformaldehyde. The tissues were sliced into 4 μm thick sections and stained with H&E. Two pathologists, who were blinded to this study, performed the evaluation. The degree of damage to the colonic samples was quantified according to the scoring system. In the colon tissues, the histological score was calculated based on three parts, including the degree of inflammation, the extent of inflammation, and the degree of crypt damage (Supplementary Table S4). This resulted in a histological score ranging from 0 (not damaged) to 10 (severely damaged).

### 4.10. Cell Culture and Transfections

Human THP-1 cells were purchased from the Cell Bank of Shanghai Institute of Biochemistry and Cell Biology, and they were then cultured in an RPMI 1640 medium (Gibco, New York, NY, USA) supplemented with 10% heat-inactivated fetal bovine serum (Gibco, Grand Island, New York, NY, USA) and 1% penicillin/streptomycin (Gibco, New York, NY, USA). The cells were cultured under a humidified 5% (*v/v*) CO_2_ atmosphere at 37 °C. THP-1 cells were stimulated with PMA (100 ng/mL) for 48 h to differentiate into macrophages (THP-M). pIRES-EGFP-Ctr and pIRES-EGFP-GRK2-WT plasmids were obtained from our lab. THPM cells were transiently transfected with different plasmids using Lipofectamine 3000 (Invitrogen). We used immunoblot analysis of whole cell lysates with specific antisera to confirm the transient expression. After transfection at 37 °C in a culture medium without serum, THP-M cells were stimulated with PGE2 (10 μM) for 30 min during the indicated time periods.

### 4.11. Immunofluorescence Assay of Mouse Colon Tissue and Cell Culture

Sections of colon tissue (6 μm) (Nest) were fixed frozen with acetone, washed in 1% PBS-Tween, permeabilized by Triton-X-100 (Beyotime, Shanghai, China) for 7 min (F4/80 without this step), then blocked with 10% goat serum (CWBIO, Taizhou, China) for 30 min, and finally incubated with the primary antibody in PBS containing 1% BSA (1:100) overnight at 4 °C. The cells were planted on a Laser confocal plate. After being washed with PBS (30 s × 3), the samples were incubated with Alexa Fluor 488 or 594 antibody IgG at 37 °C for 1 h and subsequently incubated with DAPI (10 μg/mL) for 5 min. Additionally, IgG antibody was used as a negative control to check that non-specific interaction of the primary antibody did not cause the observed staining. Images were acquired using a confocal laser-scanning microscope (Olympus, Lake Success, NY, USA).

### 4.12. Flow Cytometric Analysis

Specific antibodies against CD68, F4/80, CD86, and CD206 and isotype-matched control antibodies were purchased from e-Biosciences (San Diego, CA, USA). For cell staining, 1 × 10^6^ freshly isolated cells were incubated with fluorescent-conjugated specific antibodies against CD68, CD86, CD206, and F4/80, or isotype-matched antibodies for 30 min on ice. The cell-surface fluorescence intensity was assessed using a FACS Calibur analyzer and the CellQuest software (BD Biosciences, San Jose, CA, USA).

### 4.13. Western Blotting Analysis

Total proteins were extracted from the colon tissues or cells with different treatments in a lysis buffer (Beyotime, Shanghai, China) containing a phosphatase inhibitor cocktail (CWBIO, China) and protease inhibitor (Beyotime), and centrifuged at 14,000× *g* for 15 min at 4 °C. The supernatant was collected and a protein loading buffer (5×) was added; then, the sample was boiled for 7 min. For membrane protein preparation, the colon tissues or cells were lysed and centrifuged at 14,000× *g* for 15 min at 4 °C. The supernatant was collected and centrifuged at 100,000× *g* for 1 h at 4 °C. After removing the supernatant, the precipitated membrane protein was resuspended with 50 μL of the cell lysis buffer, and then the samples were boiled for 7 min with 10 μL of the protein loading buffer (5×). Protein concentration was determined using the BCA protein assay (Thermo, Waltham, MA, USA). The denatured protein was separated using 10% SDS-PAGE and then transferred onto a polyvinylidene fluoride (PVDF) membrane (Millipore, Burlington, MA, USA) using a semi-dry transfer system (Bio-rad, Hercules, CA, USA). After blocking with 5% non-fat powdered milk (BBI Life Sciences, Shanghai, China) for 2 h at 37 °C, the protein was detected using specific antibodies for GRK2 (1:200), EP4 (1:200), pCREB (1:150), iNOS (1:150), and Arg-1 (1:150) overnight at 4 °C, followed by HRP-conjugated anti-mouse (1:20,000) or anti-rabbit secondary antibodies (1:20,000) for 1 h at 37 °C. The protein was visualized using an enhanced chemiluminescent HRP substrate (Millipore) via chemiluminescence and was quantified using the Image J software (NIH).

### 4.14. Real-Time PCR

Total RNA was extracted from the colon tissues and THP-1 cells using Trizol reagent (Invitrogen, Carlsbad, CA, USA). Then, real-time RT-PCR was performed using the following protocol: the RNA samples were reverse transcribed to cDNA and subjected to quantitative PCR, which was performed using a Light Cycler_96 Real-Time PCR System (Roche, Basel, Swiss) with an AceQ qPCR SYBR Green Master Mix (Vazyme, Nanjing, China). The program for amplification was 1 cycle at 95 °C for 2 min, and then 40 cycles at 95 °C for 10 s, 60 °C for 30 s, and 95 °C for 10 s. The PCR data of each gene were normalized to the housekeeping gene glyceraldehyde 3-phosphate dehydrogenase (GAPDH) expression and were quantified using the ΔΔCT method. The primer sequences used in this study are listed in the Supplementary Table S5.

### 4.15. Statistical Analysis

All results were analyzed using the Prism version 7.00 software (GraphPad Software, San Diego, CA, USA). A two-way ANOVA with Dunnett’s Multiple Comparison test was applied to analyze the significance between multiple groups, and an unpaired Student’s *t*-test was applied to analyze the significance between two groups. Statistical significance was indicated by a *p*-value of <0.05. The data were expressed as the mean ± SEM of three independent experiments.

## 5. Conclusions

In conclusion, we provide new evidence demonstrating that paroxetine attenuates symptoms of DSS-induced colitis in a mouse model. The group treated with paroxetine had lower weight loss, lower disease activity index, and lower pathological score. Additionally, pro-inflammatory cytokines in peripheral blood significantly reduced. Our results regarding peritoneal macrophages showed that paroxetine could block GRK2-EP4 interaction to adjust the ratio of M1/M2 by influencing the levels of cAMP and p-CREB. These results suggest that GRK2 may act as a novel therapeutic target for UC by regulating macrophage polarization.

## Data Availability

Data is contained within the article or Supplementary Material. The raw data supporting the conclusions of this manuscript will be made available by the corresponding author, without undue reservation.

## Supplementary Materials

The following supporting information can be downloaded at https://www.mdpi.com/article/10.3390/ph16050664/s1. Figure S1: Paroxetine had therapeutic effect in mice with DSS-induced colitis.; Table S1: Baseline Clinical Characteristics; Table S2: Selected laboratory examination characteristics. Table S3: Disease activity index (DAI) criteria. Table S4: Histological scoring system for colonic samples. Table S5: Primer sequence used for qRT-PCR.

## Abbreviations

cAMP: cyclic adenosine monophosphate; CD, Crohn’s disease; CIA, collagen-induced arthritis; CREB, cyclic AMP responsive element-binding protein; DAI, disease activity index; EP4, prostaglandin E2 receptor 4; FBS, fetal bovine serum; GPCRs, G protein-coupled receptors; GRK2, G protein-coupled receptor kinase 2; HBSS, Hank’s salt solution; H&E, hematoxylin and eosin; IBD, inflammatory bowel disease; IL, interleukin; LPMCs, lamina propria mononuclear cells; memb, membrane; pae, paeoniflorin; PBS, phosphate-buffered saline; PGE2, prostaglandin E2; PVDF, polyvinylidene fluoride; SASP, salicylazosulfapyridine; SSRI, selective serotonin reuptake inhibitors; THP-1, human acute monocytic leukemia cells; TNF-α, tumor necrosis factor α; UC, ulcerative colitis.
