# Supplementary material for: GRK2 Mediates Macrophage Polarization by Regulating EP4-cAMP-pCREB Signaling in Ulcerative Colitis and the Therapeutic Effect of Paroxetine on Mice with DSS-Induced Colitis
Source: Pharmaceuticals (Basel). 2023 Apr 28;16(5):664. doi: 10.3390/ph16050664 (PMC10223763; doi:10.3390/ph16050664)
Supplement: Supplementary file 1 [file pharmaceuticals-16-00664-s001.zip › pharmaceuticals-2244175-supplementary.pdf]

Supplementary Table 1

Baseline Clinical Characteristics.

Parameter	Control	Ulcerative colitis	*P*-value
	22	22	
**Gender**			
Female	12	7	＞0.05
Male	10	15	
**Age(years)**			
≤65	13	19	＞0.05
＞65	9	3	
**BMI**			
＜25	13	20	＞0.05
≥25	9	2	
**Smoke**			
Yes	8	4	＞0.05
No	14	18	
**Alcohol**			
Yes	5	1	＞0.05
No	17	21	
**Mayo score**			
≤2(Normal)	22	3	＞0.05
3-5(Mild)	0	5	
6-10(Moderate)	0	6	
11-12(Severe)	0	8	

Supplementary Table 2

Selected laboratory examination characteristics.

Parameter	Overall	Control	Ulcerative colitis
n	44	22	22
**WBC(*10^9/L)**	7.36±1.67	6.29±1.41	8.98±3.02
**Hb(g/L)**	118.5±9.54	123.5±11.32	110.5±16.04
**ESR(mm/h)**	22.97±12.57	12.05±6.34	35.86±8.69
**CRP(mg/l)**	27.54±4.19	3.36±1.21	40.38±7.82
**PT(s)**	12.2±1.22	11.7±1.01	13.32±1.83

Supplementary Table 3

Disease activity index (DAI) criteria.

Grade	Weight loss/%	Stool consistency	Rectal bleeding
0	0	Normal	N/A
1	1-5	Mild soft	-
2	5-10	Soft and wet	Hemoccult positive
3	10-20	Half loose stool	-
4	>20	Loose stool	Gross bleeding

Supplementary Table 4

Histological scoring system for colonic samples.

Grade	Severity of inflammation	Extent of inflammation	Crypt damage
0	None	None	None
1	Mild	Mucosa	1/3 damaged
2	Moderate	Mucosa and submucosa	2/3 damaged
3	Severe	Transmural	Crypt loss by surface epithelium present
4	-	Loose stool	Both crypt and surface epithelium lost

Supplementary Table 5

Primer sequence used for qRT-PCR.

Gene	Orientation	Sequence (5′–3′)
Human IRF5	Forward	GAC TTC CGC CTC ATC TAC GA
	Reverse	CCT CTG CAG CTC TTC CTC TT
Human IRF4	Forward	CAC CAT GAC AAC GCC TTA CC
	Reverse	CAT TTT CAC AAG CTG GGC CT
Human GAPDH	Forward	GAA AGC CTG CCG GTG ACT AA
	Reverse	GCC CAA TAC GAC CAA ATC AGA G
Mouse IRF5	Forward	ACA TGT TGC CTT TGA CGG AC
	Reverse	AGA AAC GCT GCT TGT CAC TG
Mouse IRF4	Forward	AAC TAC ATG ATG CCA CCC CA
	Reverse	TAT GCT TGG CTC AAT GGG GA
Moue GAPDH	Forward	ACC CTT AAG AGG GAT GCT GC
	Reverse	CCC AAT ACG GCC AAA TCC GT

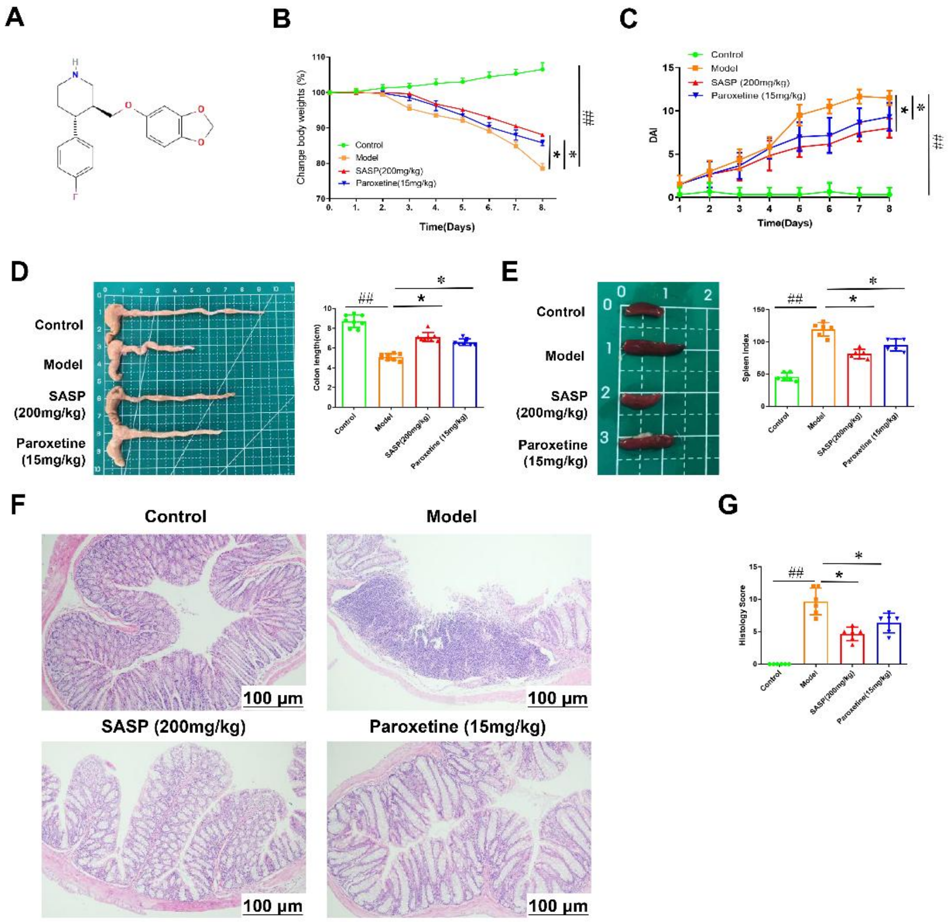

Supplementary Figure1. Paroxetine had therapeutic effect in mice with DSS-induced colitis. (A) Chemical structure of paroxetine. (B) Body weight changes and (C) Disease activity index (DAI) evaluations during the disease process. (D) Macroscopic photographs and length of the colons. (E) Macroscopic photographs of the spleen and spleen index. (F) Representative H&E staining images of colon tissue (scale bar, 100 μm), and (G) Histological scores of colon tissue. The data are presented as the means ± SEM (n = 6) and statistical analysis was performed using one-way ANOVA with Dunnett's Multiple Comparison test. Significant differences were indicated as #P < 0.05, ##P < 0.01 vs. Control group; *P < 0.05 vs. paroxetine group; *P<0.05 vs. SASP group.
